# An annotated street view image dataset for automated road damage detection

**DOI:** 10.1038/s41597-024-03263-7

**Published:** 2024-04-22

**Authors:** Miao Ren, Xianfeng Zhang, Xiaobo Zhi, Yuanjia Wei, Ziyuan Feng

**Affiliations:** Institute of Remote Sensing and Geographic Information System, Peking University, 5 Summer Palace Road, Beijing, 100871 China

**Keywords:** Natural hazards, Sustainability

## Abstract

Road damage is a great threat to the service life and safety of roads, and the early detection of pavement damage can facilitate maintenance and repair. Street view images serve as a new solution for the monitoring of pavement damage due to their wide coverage and regular updates. In this study, a road pavement damage dataset, the Street View Image Dataset for Automated Road Damage Detection (SVRDD), was developed using 8000 street view images acquired from *Baidu Maps*. Based on these images, over 20,000 damage instances were visually recognized and annotated. These instances were distributed in five administrative districts of Beijing City. Ten well-established object detection algorithms were trained and assessed using the SVRDD dataset. The results have demonstrated the performances of these algorithms in the detection of pavement damages. To the best of our knowledge, SVRDD is the first public dataset based on street view images for pavement damages detection. It can provide reliable data support for future development of deep learning algorithms based on street view images.

## Background & Summary

As the most fundamental and widely used transportation infrastructure, highways ensure the rapid and efficient flow of people and goods. They also play a key role in economic and social development. However, under repeated vehicle loads and harsh environmental conditions, road surface structures undergo aging and deterioration, eventually leading to road damage. This has a severe impact on road performance^1^. Therefore, the rapid and precise monitoring of road pavement damage and its distribution play a crucial role in extending the service life of highway roads.

Conventional road damage detection techniques typically depend on manual visual detection and vehicle-mounted road Pavement Monitoring Systems (PMS). Such manual-based approaches are greatly influenced by the experience of road maintenance personnel, who primarily employs ground measurements and visual assessments to detect road pavement health. These techniques are often time-consuming, inefficient, and traffic-disruptive, consequently making them unsuitable for monitoring extensive road pavements^2^. PMS equipped with multiple sensors can acquire comprehensive road information, yet they are associated with high acquisition and operating costs. Hence, they are generally only employed for high-level road surfaces and have a low efficiency^3^.

**Fig. 1:**
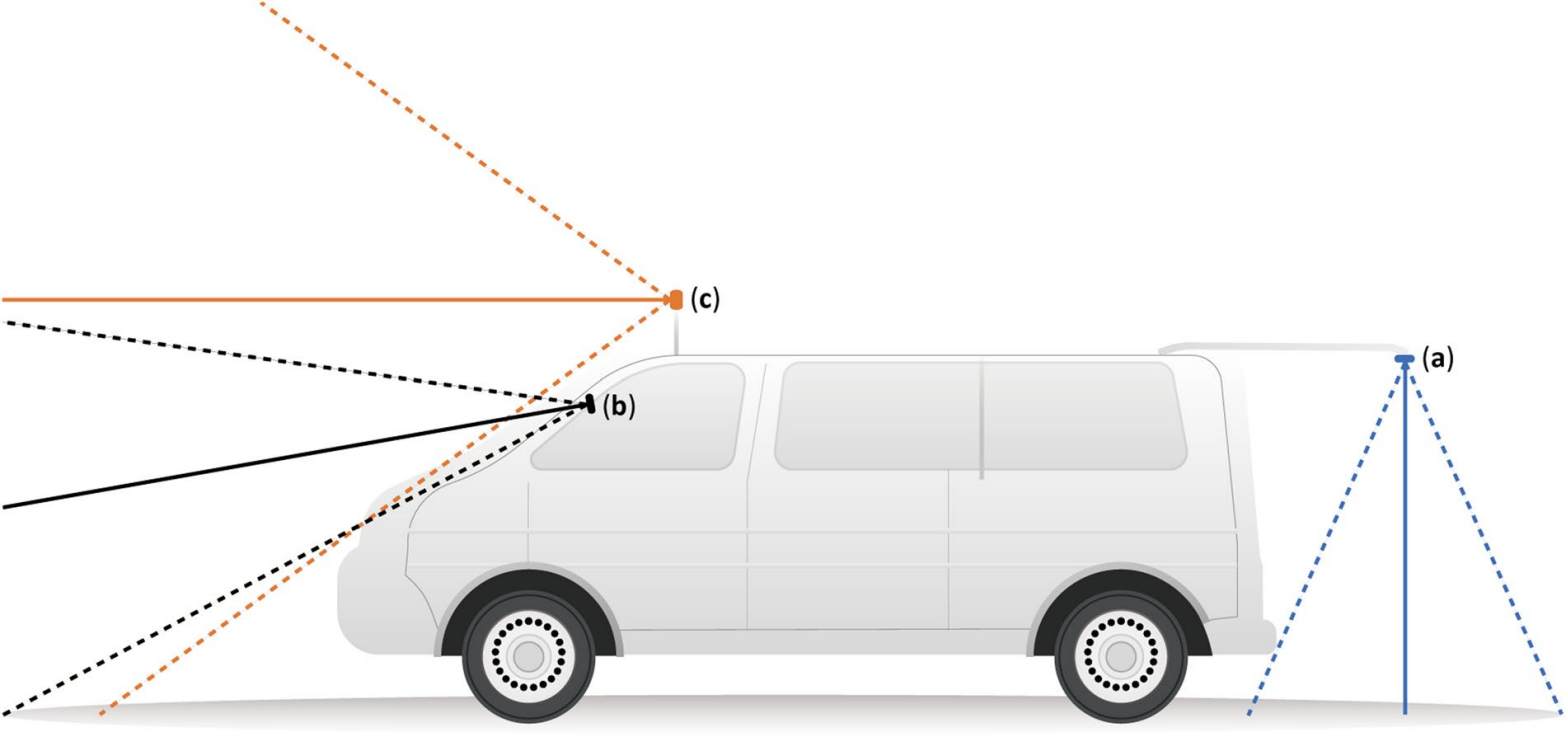
Camera set-ups, angles, and ranges under vehicle platform for different views of road damage detection datasets. (**a**) top-down view; (**b**) wide view; (**c**) street view.

With the development of computer vision and deep learning, image classification, object detection, and segmentation techniques have been widely employed in the detection of road pavement damages. Currently, the image data for road pavement damage detection predominantly originates from ground-based platforms, encompassing top-down view, wide view, and street view perspectives. The top-down view images are usually captured by vehicle-mounted or handheld cameras or smartphones, positioned a few meters above the ground at a perpendicular angle to the road surface. The wide view images are usually taken from smartphones installed either on the dashboard or windshield of vehicles, capturing the road conditions from a forward and slightly downward angle. The street view images often consist of the front view of street view images, providing insights of road conditions. The set-ups, angles, and ranges of cameras under vehicle platform for these three perspectives are illustrated in Fig. 1.

The present public datasets for road pavement damage detection are limited to top-down and wide view images. Table 1 reports an overview of the major datasets available^4–18^. Crack Forest Dataset (CFD) is a representative dataset for pavement damage segmentation. Such datasets use smartphones and vehicle-mounted cameras to capture images of road surfaces from a top-down view and subsequently annotate pavement damages on a pixel-by-pixel basis. However, they only distinguish between cracks and background road categories, while other damage categories are not considered. Moreover, the amount of data is small and the image resolution is inconsistent^4–11^. The German Asphalt Pavement Distress (GAPs) v1^12^ and GAPs v2^13^ datasets use vehicle-mounted CCD imagers to capture the images of road surfaces from a top-down view with an image array of 1920 × 1080. Six road damage categories are annotated with bounding boxes, making them suitable for the object detection of road damage. In particular, the GAPs 10 m^14^ dataset, released in 2021, contains 20 high-resolution images (5030 × 11,505 pixels) covering 200 m of asphalt pavement of different road categories. Totally 22 categories of objects and damage instances at the pixel level are annotated, facilitating the fine-grained segmentation of road damage. The Road Damage Dataset (RDD) series datasets^15–18^, which was recently updated from 2018 to 2022, uses smartphones installed on windshields to capture wide view road images and annotates four damage categories (*i.e*., longitudinal cracks, transverse cracks, alligator cracks, and potholes). Furthermore, the Global Road Damage Detection Challenge (GRDDC) has attracted much attention in research on road damage detection^19^. Despite the great progress made by these studies on the application of deep learning algorithms for the detection of road pavement damages, they are usually associated with high acquisition costs, varying image resolutions, and restricted image views, which imped the practical application of the associated models.

**Table 1:** Public datasets used for the detection of road pavement damage.

Dataset	Images	Resolution	View captured	Annotation-level	Annotated category
CFD^4,5^	118	480 × 320	Top-down view	Pixel	2 (good and distressed road)
CrackTree206^6^	206	800 × 600	Top-down view	Pixel	2 (crack and background)
CrackWH100^7^	100	512 × 512	Top-down view	Pixel	2 (crack and background)
CrackLS315^7^	315	512 × 512	Top-down view	Pixel	2 (crack and background)
DeepCrack^8^	537	544 × 384	Top-down view	Pixel	2 (crack and background)
Crack500^9^	500	2000 × 1500	Top-down view	Pixel	2 (crack and background)
CrackNJ156^10^	156	512 × 512	Top-down view	Pixel	2 (crack and background)
CrackSC^11^	197	320 × 480	Top-down view	Pixel	2 (crack and background)
GAPs v1^12^	1969	1920 × 1080	Top-down view	Bounding box	6 (cracks, potholes, inlaid patches, applied patches, open joints, and bleedings)
GAPs v2^13^	2468	1920 × 1080	Top-down view	Bounding box	6 (cracks, potholes, inlaid patches, applied patches, open joints, and bleedings)
GAPs 10 m^14^	20	5030 × 11,505	Top-down view	Pixel	22
RDD2018^15^	9053	600 × 600	Wide view	Bounding box	8 (linear crack, longitudinal, wheel mark part; linear crack, longitudinal, construction joint part; linear crack, lateral, equal interval; linear crack, lateral, construction joint part; alligator crack; rutting, bump, pothole, separation; cross walk blur; white line blur)
RDD2019^16^	13,135	600 × 600	Wide view	Bounding box	9 (linear crack, longitudinal, wheel mark part; linear crack, longitudinal, construction joint part; linear crack, lateral, equal interval; linear crack, lateral, construction joint part; alligator crack; rutting, bump, pothole, separation; cross walk blur; white line blur; utility hole)
RDD2020^17^	26,620	600 × 600, 720 × 720	Wide view	Bounding box	4 (longitudinal cracks, transverse cracks, alligator cracks, and potholes)
RDD2022^18^	47,420	512 × 512, 600 × 600, 720 × 720, 3650 × 2044	Wide view, extra-wide view, top-down view	Bounding box	4 (longitudinal cracks, transverse cracks, alligator cracks, and potholes)

Street view images are geotagged images collected by map service providers (*e.g*., *Google Maps* and *Baidu Maps*) through street view imaging systems along roads from multiple viewing angles. These images are then processed and maintained according to the standard methods^20^. Street view images can accurately depict the urban physical environment^21^ and have been employed in numerous research applications, including the estimation of poverty, violent crime, health behaviour, and travel patterns^22,23^. The front view of street view images provides insights of road conditions. These images, which are collected by state-of-the-art devices, have the advantages of low additional costs, easy accessibility, regular updates, and rich data. Thus, street view images offer new data sources for the detection of road pavement damages. Table 2 summarizes the current street view image datasets and their application in the existing studies for the detection of road damage^24–31^. The datasets used in the studies vary in terms of quantity, spatial resolution, and annotated damage category, and are not publicly available at all. Classifying pavement damage solely at the image level is inadequate, as it can only indicate the presence or absence of damages^25,29^. While segmenting pavement damage at the pixel level provides detailed information on the shape and size, current studies predominantly focuses only on the presence or absence of damages and is restricted by a limited image dataset^24,28^. Some road damage detection studies relying on bounding boxes explore few categories of damages, failing to meet industry requirements^30^. Alternatively, some damage categories may not be suitable for street view image scenarios^26,27^. Moreover, some datasets have a relatively limited coverage^31^. Although the models proposed in these studies have made progress in monitoring road damages, their performance is only validated on self-built datasets and there is a lack of training and testing on unified public datasets. This makes it challenging to fairly evaluate and compare the performances of various models.

**Table 2:** Reported street view image datasets and their application in the detection of road damage.

Dataset	Images	Resolution	Source	Annotation-level	Annotated category	Methods	Availability
Chacra and Zelek^24^	250	640 × 640	Google Street View	Pixel	2 (crack and background)	Linear SVM	Private
Ma *et al*.^25^	711,520	640 × 224	Google Street View	Image	3 (poor, fair, and good)	FC-CNN	Private
Majidifard *et al*.^26^	7237	640 × 640	Google Street View	Bounding box	9 (reflective crack, transvers crack, block crack, longitudinal crack, alligator crack, sealed reflective crack, lane longitudinal crack, sealed longitudinal crack, and pothole)	YOLOv2, Faster R-CNN	Private
Lei *et al*.^27^	19,665	1024 × 512	Baidu Maps	Bounding box	8 (deformation, pothole, loose, net-crack, cracks, patched-pothole, patched-net, and patch-crack)	YOLOv3	Private
Zhang *et al*.^28^	400	1024 × 512	Baidu Maps	Pixel	2 (crack and background)	Deeplabv3+	Private
Maniat *et al*.^29^	27,000	200 × 200	Google Street View	Image	2 (cracked and not cracked)	VGG-16	Private
Maniat *et al*.^29^	67,000	250 × 250	Google Street View	Image	5 (not cracked, longitudinal crack, transverse crack, alligator crack, and not pavement)	VGG-16	Private
Shu *et al*.^30^	400	512 × 512	Baidu Maps	Bounding box	3 (transverse, longitudinal, and alligator cracks)	YOLOv5l	Private
Ren *et al*.^31^	2900	1024 × 1024	Baidu Maps	Bounding box	7 (longitudinal crack, transverse crack, alligator crack, pothole, manhole cover, longitudinal patch, and transverse patch)	YOLOv5s-M	Private

Compared to the top-down and wide view images, the street view images stand out with distinct characteristics when utilized for road pavement damage detection. The top-down and wide view images cover only the captured areas since they are captured privately. If a trained model is applied to the actual area to be detected, images of the study area need to be acquired. The street view images are captured by map service providers using specialized equipment and are guaranteed in terms of image quality. The data covers almost all cities in the world, is publicly available for download, and updated regularly. The model trained using the road damage dataset of annotated street view images can be easily used in street view images of other areas. Also, these three views datasets can be used as data for domain adaptation studies with each other. And the wide view and street view images have similar perspectives and the same complex road background, which can be better used for the study of pavement damage domain adaptation. Table 3 shows the comparison of the attributes of the three views image datasets.

**Table 3:** Attributes of top-down view, wide view, and street view image dataset for road pavement damage detection.

Attributes	Top-down view	Wide view	Street view
Platform	Vehicle, Handheld	Vehicle	Vehicle
Device	Camera, Smartphone	Smartphone	Street view imaging systems
Position	A few meters above the road	On the dashboard/windshield of the vehicle	On the roof of the vehicle
Angle	Downward, perpendicular to the road	Forward, slightly downward to the road	Directly forward, parallel to the road
Background	None	Complex road environment	Complex road environment
Acquisition	Privately obtained	Privately obtained	Publicly downloadable
Coverage	Captured areas	Captured areas	Global coverage
Updatable	No update	No update	Regular update

In this study, we propose the Street View Image Dataset for Automated Road Damage Detection (SVRDD), a dataset based on street view images for the detection of road pavement damage. To the best of our knowledge, SVRDD is the first public dataset based on street view images for pavement damages detection. It comprises a total of 8000 street view images from Dongcheng, Xicheng, Haidian, Chaoyang, and Fengtai Districts of Beijing City, encompassing a variety of urban road types and pavement conditions. The dataset comprehensively annotates pavement damages at the bounding box level, encompassing a total of 20,804 annotated instances. In terms of both the number of images and the damage instances, the SVRDD dataset stands out among current datasets for road damage detection based on street view imagery, at both bounding box and pixel annotation levels. The categories of pavement damage addressed include six damage categories and one confusing non-concrete pavement, namely, longitudinal crack, transverse crack, alligator crack, pothole, longitudinal patch, transverse patch, and manhole cover. From an application perspective, pavement damage categories in SVRDD are more relevant to the transportation industry sector and are well-suited for detection in street view imagery. Simultaneously, the inclusion of manhole cover annotations can significantly enhance the detection of pothole^16,31^. SVRDD provides bounding box annotations in two formats (*i.e*., Pascal VOC and YOLO) to facilitate the easy usage of the datasets. The backgrounds of the street view images in SVRDD include pedestrians, vehicles, buildings, viaducts, trees, and their shadows. The images were collected under multiple seasons and weather and lighting conditions. In order to evaluate SVRDD, we trained and tested ten well-established object detection algorithms using this dataset. We subsequently analysed the performance of the dataset with varying numbers of training images and evaluated the impact of the training subsets association from different districts on the model training, to assist users in utilizing the dataset. Additionally, some potential extensions for the SVRDD dataset were further analysed, which opens the door for further research.

## Methods

**Fig. 2:**
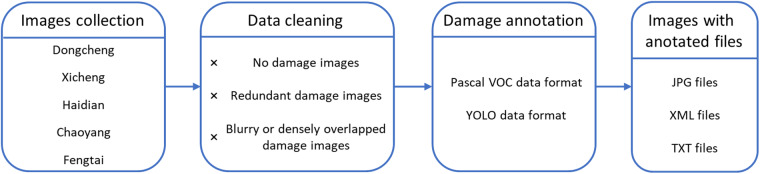
Flowchart of the generation process of SVRDD.

The creation of the SVRDD dataset includes three key steps, namely, image collection, data cleaning, and damage annotation (Fig. 2).

### Images collection

A total of 844,432 street view images of Beijing City were acquired from *Baidu Maps*^32^. Notably, the use of *Baidu Maps* street view images must comply with its terms and conditions^33^. First, road location information was obtained using the Open Street Map (OSM) road network data, which was converted to the BD09 coordinate system used in the *Baidu Maps*. A sampling point was then generated every five meters along a road network, and the coordinates of the sampling points and other parameters (*e.g*., image width, height, and viewing angle) were input into the *Baidu Maps* API to download the street view images. Two types of images, with pitch angles of 0° and 45°, respectively, were obtained for each sample location. These images were vertically concatenated to obtain a complete front-view street view image for a given location. Each image has a size of 1024 × 1024 pixels. The street view images used for the dataset were mainly captured in 2019 and 2020.

### Data cleaning

The large number of obtained street view images ensures a wide distribution of damage features. However, as street view images are not specifically designed for the detection of road damage, data cleaning was required to guarantee the quality of the pavement damage dataset. The data cleaning process was performed following three steps: i) removal of images without damage; ii) considering the high sampling frequency of street view images and the minimal road damage differences between adjacent images, redundant images were also removed; and iii) deletion of images with either blurry or densely overlapped instances of damage. Considering the annotation workload and district area, we ultimately obtained 8000 street view images for the detection of road pavement damage, with 1000 images from Dongcheng and Xicheng districts, respectively, and 2000 images from Haidian, Chaoyang, and Fengtai districts, respectively.

### Damage annotation

All the selected images were manually annotated using *LabelImg* with object bounding boxes. The annotated damage categories include longitudinal cracks, transverse cracks, alligator cracks, potholes, longitudinal patches, and transverse patches. Due to the potential misclassification of pothole and manhole cover^16,31^, we added a category for manhole covers. The annotation process was done by three trained annotators, and the results from each annotator were cross-checked using the other two annotators. Pascal VOC and YOLO were included as the annotation formats. For the Pascal VOC format, data is stored in .xml files, and the position of a bounding box is represented as (x_min_, y_min_, x_max_, y_max_), with (x_min_, y_min_) and (x_max_, y_max_) as the top-left and bottom-right coordinates, respectively. For the YOLO format, data is stored in.txt files, and the position of a bounding box is represented as (x, y, w, h), with (x, y), w, and h as the centre coordinate, width, and height of the bounding box, respectively.

### Image properties

**Fig. 3:**
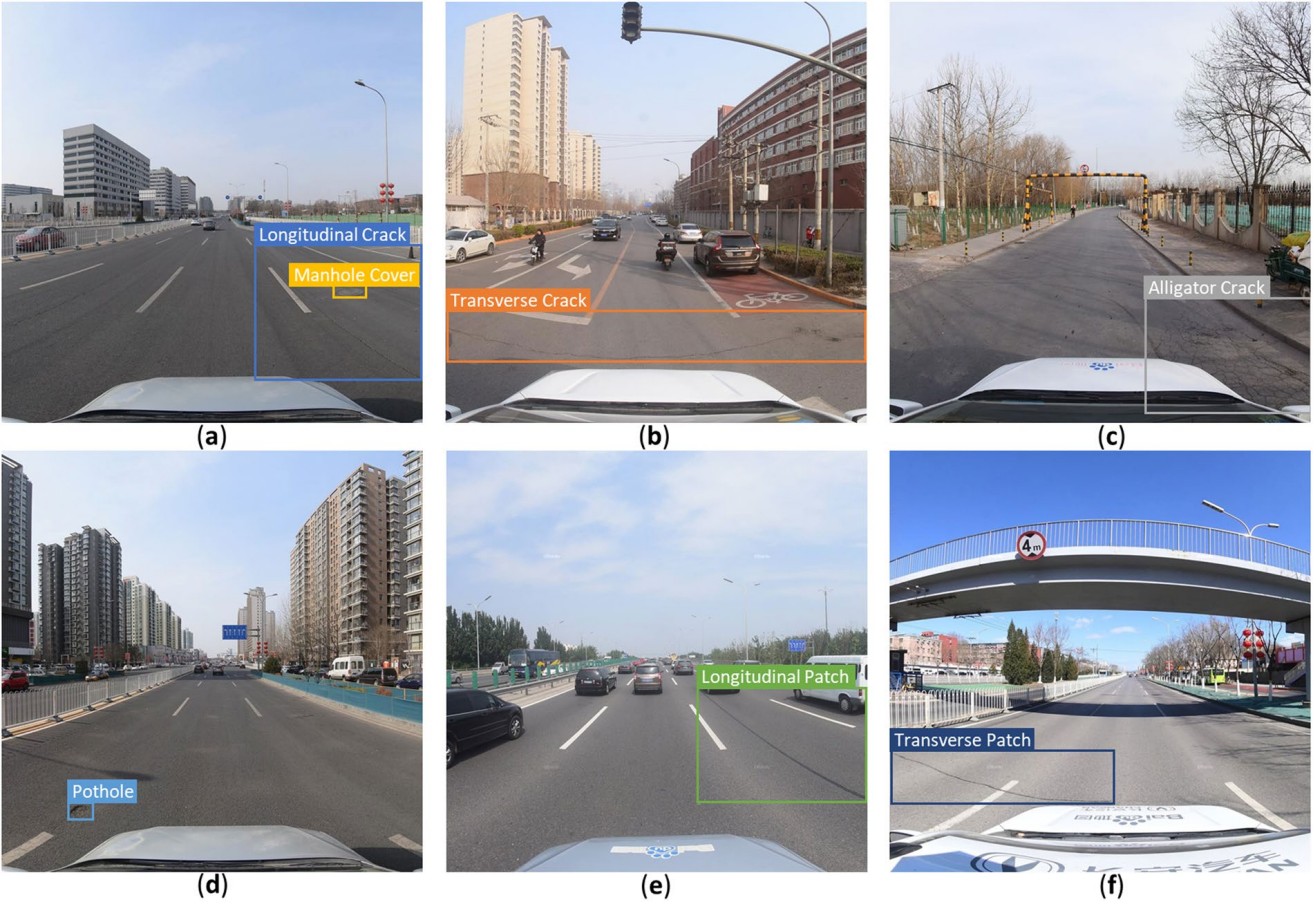
Examples of different damage categories included in the SVRDD dataset. (**a**) longitudinal crack and manhole cover; (**b**) transverse crack; (**c**) alligator crack; (**d**) pothole; (**e**) longitudinal patch; (**f**) transverse patch.

Statistical analysis was performed to determine the distribution and properties of the SVRDD dataset. Figure 3 presents some example images from the dataset with different damage category annotations. The background of the images contains pedestrians, vehicles, buildings, viaducts, trees, and their shadows. The images were collected under different seasons and weather and lighting conditions. These varying conditions bring challenges to the detection of road damage from the street view images.

**Fig. 4:**
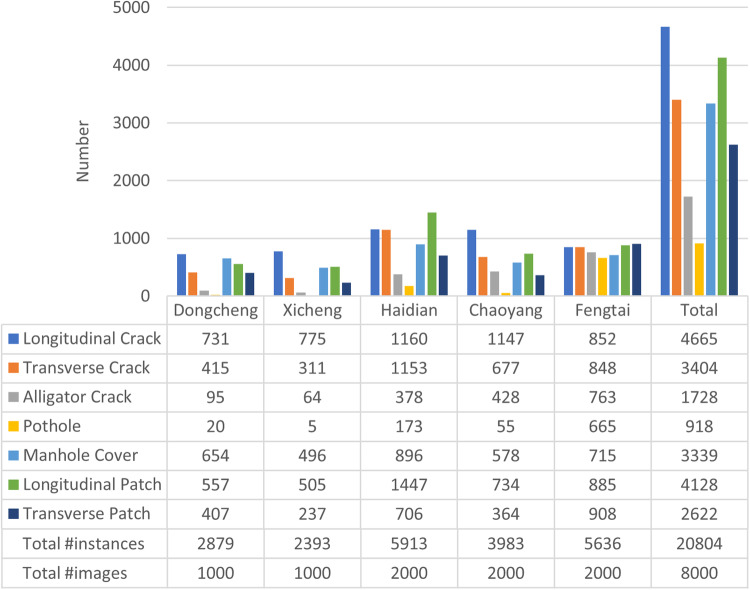
Statistics of the number of damage instances included in the SVRDD dataset.

The number of damage instances of each category in the SVRDD dataset and the key statistics of each district are illustrated in Fig. 4. The 8000 images in the SVRDD dataset offer a total of 20,804 damage annotation instances. Among the six damage categories, the number of potholes and alligator cracks is relatively low, as these two damage types emerge after severe road aging. Among the five districts, Chaoyang District exhibits the lowest average number of damage instances.

**Fig. 5:**
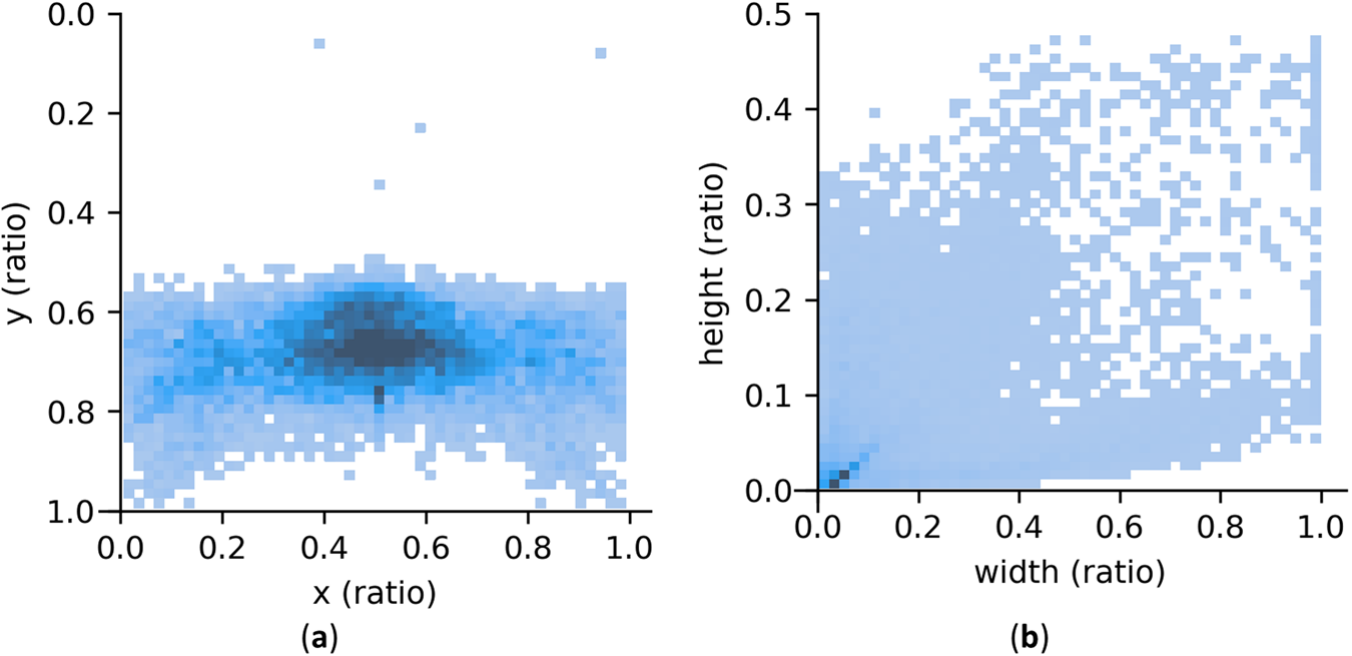
Position and shape statistics of the damage instances. (**a**) distribution of the central point coordinate; (**b**) distribution of the ratio between the height and width.

The position and shape statistics of the damage instances in the SVRDD dataset, namely, the central point coordinates and height–width distributions are presented in Fig. 5. The central points of the damage instances are primarily concentrated in the lower half of the images, while the upper half of the images generally represent the background. Due to the perspective effect, the road surfaces located in the upper half of the images are narrower, making it challenging to identify damage. The height–width distribution of the damage instances reveals that the damage width can span the entire image, while the length is at most equal to half of the image.

**Fig. 6:**
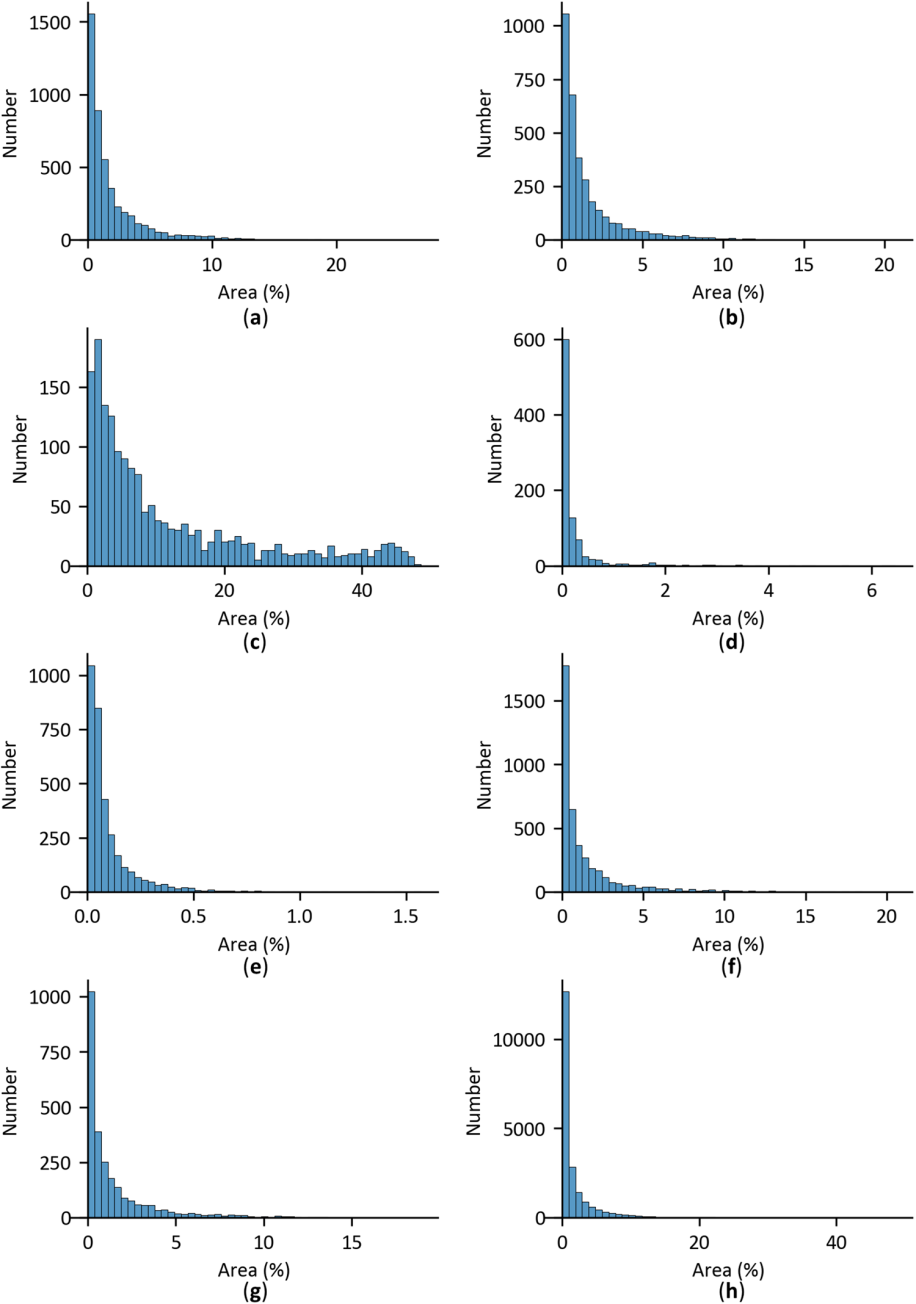
Area statistics of damage instances. (**a**) longitudinal crack; (**b**) transverse crack; (**c**) alligator crack; (**d**) pothole; (**e**) manhole cover; (**f**) longitudinal patch; (**g**) transverse patch; (**h**) total.

The area statistics of damage instances in the SVRDD dataset, which are calculated as the ratio of the area of damage instances to the area of images, are illustrated in Fig. 6. The area distributions of longitudinal cracks, transverse cracks, longitudinal patches, and transverse patches are approximately the same, and concentrated within 10% of the image area. Alligator cracks have a relatively large area, with the highest value reaching 50% of the image area. Pothole and manhole cover are determined to have smaller areas, mostly less than 0.5% of the image area. The results demonstrate there to be significant differences in the area of damage instances. Thus, the object size needs to be considered when constructing a deep learning network for road damage detection.

## Data Records

The SVRDD dataset has been published in the Zenodo repository^34^. Its data structure and format are described in the following.

**Fig. 7:**
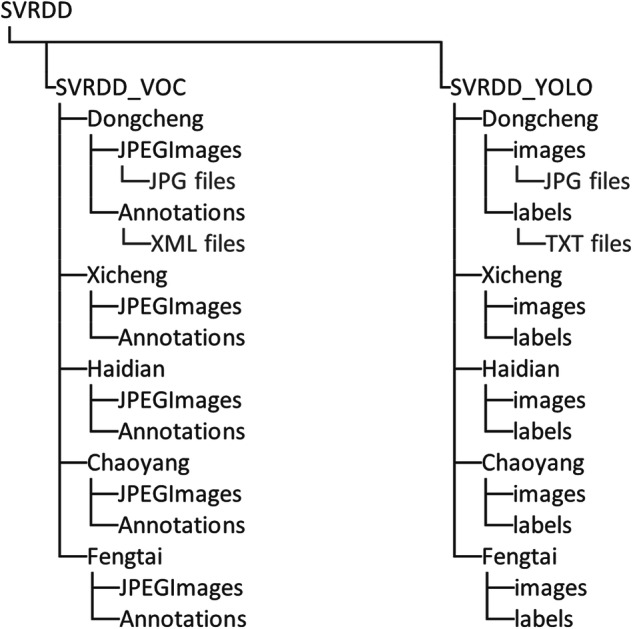
Directory structure of the SVRDD dataset (the file types in folders are exemplified in the ‘Dongcheng’ folder).

The dataset includes two folders, namely, ‘SVRDD_VOC’ and ‘SVRDD_YOLO’. The ‘SVRDD_VOC’ organizes the data in Pascal VOC format and contains the ‘JPEGImages’ folder with the street view images of all the districts of Beijing City, as well as the ‘Annotations’ folder which contains the corresponding bounding box annotation files in .xml format. The ‘SVRDD_YOLO’ organizes the data in YOLO format. It contains the ‘images’ folder with the street view images of all districts and the ‘labels’ folder with the corresponding bounding box annotation files in.txt format. The directory structure for the SVRDD dataset is shown in Fig. 7.

Each image filename consists of the image serial number PID, horizontal coordinate X of the shooting position, and vertical coordinate Y of the shooting position, separated by ‘_’. PID is the serial number of the image provided by *Baidu Maps* and the horizontal and vertical coordinates of the image shooting position are the corresponding coordinates in the *Baidu* BD09 coordinate system.

## Technical Validation

The technical validation of the SVRDD dataset evaluates its applicability in the construction of deep learning models for the detection of road damage based on street view images. In the technical validation, the dataset was randomly split into a training set of 6000 images, validation set of 1000 images, and testing set of 1000 images at a ratio of 6:1:1. The proportions of images from each district in the three sample sets are consistent with the overall image proportions of each district.

### Performance of object detection algorithms using SVRDD

The SVRDD dataset was used to train and evaluate the performance of ten mainstream object detection algorithms, including Faster R-CNN^35^, Cascade R-CNN^36^, Dynamic R-CNN^37^, RetinaNet^38^, FCOS^39^, ATSS^40^, YOLOv3^41^, YOLOF^42^, YOLOv5^43^, and YOLOX^44^. The experimental hardware configuration includes an Intel (R) Xeon (R) Silver 4116 CPU, 128 GB RAM, and four NVIDIA GeForce 1080Ti GPUs. The open-source object detection library MMDetection^45^ was used as the implementation platform for the algorithms. For the parameter settings, the batch size was set to 16, the stochastic gradient descent algorithm was selected to optimize the learning rate, the momentum was set to 0.9, the weight decay coefficient was 0.0001, and a warm-up method was employed to initialize the learning rate. Table 4 reports the performance comparison of these models on the SVRDD testing set. Results indicate that YOLOv5, YOLOX, and Cascade R-CNN demonstrated superior performances on the SVRDD dataset. Among them, YOLOv5 exhibited the best detection performance with a *F1*-score of 0.709 and a *mAP*@0.5 of 0.733. The YOLOv5 network attends to characterize object features at four scales with strides of 8, 16, 32, and 64, while thoroughly fusing features between different layers, significantly enhancing the detection of pavement damage with substantial variations in size. It can be seen in Table 4 that YOLOX trailed closely with a *F1*-score of 0.691 and a *mAP*@0.5 of 0.703, notching the highest *mAP*@0.5:0.95 of 0.420. Next is the Cascade R-CNN, which having the largest parameters and FLOPS, recorded a *F1*-score of 0.664 and a *mAP*@0.5 of 0.674. Following is Dynamic R-CNN, and the most classic object detection algorithm Faster R-CNN and YOLOv3 also achieved good performance. The remaining detection algorithms (*i.e*., RetinaNet, FCOS, ATSS, and YOLOF) also achieved *F1*-score and *mAP*@0.5 values nearly reaching 0.6. These results reveal the effectiveness of the SVRDD dataset with deep learning algorithms for the detection of road damage in street view images.

**Table 4:** Performance comparison of different mainstream object detection algorithms.

Methods	Parameters	FLOPs	Precision	Recall	*F1*-Score	*mAP*@0.5	*mAP*@0.5:0.95
Faster R-CNN	41.379 M	274.4 G	0.586	0.662	0.622	0.649	0.363
Cascade R-CNN	69.170 M	331.8 G	0.657	0.671	0.664	0.674	0.403
Dynamic R-CNN	41.753 M	276.5 G	0.625	0.653	0.639	0.660	0.379
RetinaNet	36.454 M	264.2 G	0.547	0.637	0.589	0.604	0.309
FCOS	32.127 M	251.9 G	**0.806**	0.450	0.578	0.602	0.322
ATSS	32.127 M	258.0 G	0.763	0.536	0.630	0.636	0.349
YOLOv3	61.556 M	139.9 G	0.767	0.554	0.643	0.640	0.279
YOLOF	42.478 M	122.6 G	0.608	0.573	0.590	0.563	0.298
YOLOv5	12.331 M	16.2 G	0.752	0.671	**0.709**	**0.733**	0.417
YOLOX	25.284 M	73.5 G	0.675	**0.707**	0.691	0.703	**0.420**

### Performance with varying training set sizes

**Fig. 8:**
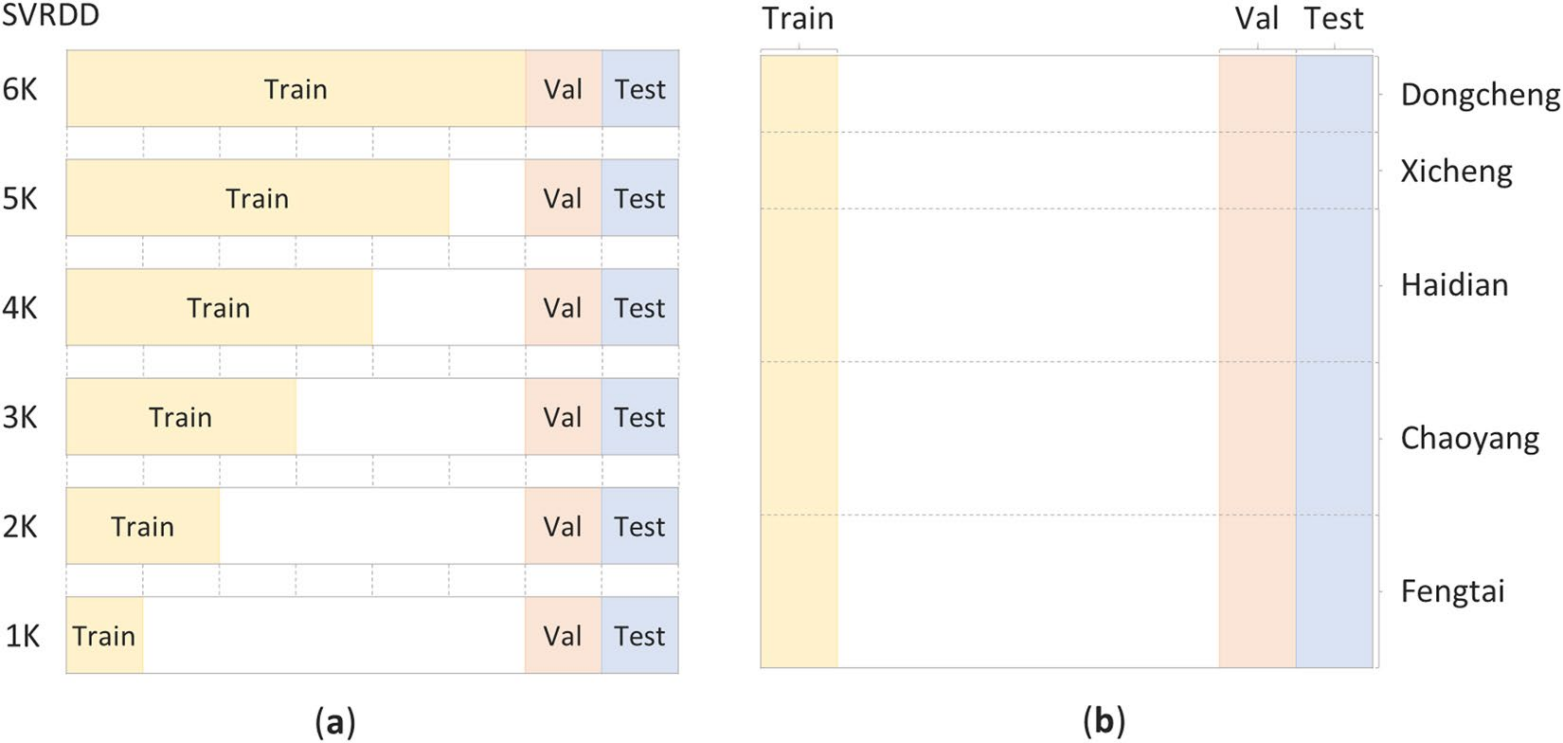
Dataset splitting for different training set sizes (**a**) and the proportion of images for each district in the SVRDD1K dataset (**b**).

The model performance was investigated using sub-datasets with different numbers of training images. The datasets shared the same validation and testing sets as the original SVRDD dataset, while the number of images in the training set were varied. Figure 8 presents the division of the datasets. SVRDD6K is the original SVRDD dataset, with 6000 images in the training set, SVRDD5K is derived from SVRDD6K by removing 1000 images from the training set based on the image proportions of each district, and so forth for the remaining datasets. Notably, the proportions of images in each district in the training sets of these datasets remain consistent with the original proportions of images in each district.

As YOLOv5 presents the best performance in the comparative experiments, it was trained and tested on these six datasets (*i.e*., 6K to 1 K) (Table 5). As the number of training images decreased within a certain range, the model accuracy also decreased. Compared to SVRDD6K, when the number of training samples is 5000, 4000, 3000, 2000, and 1000, the model’s *F1*-score decreased by 0.28%, 0.42%, 1.97%, 8.04%, and 23.13%, and the model’s *mAP*@0.5 decreased by 0, 2.18%, 4.50%, 11.60%, and 29.33%, respectively. The model performance is approximately equal for the SVRDD6K and SVRDD5K datasets, with only a slight decrease in the *F1*-score under the latter. This suggests that the model’s performance is significantly influenced by the dataset size. However, as the dataset size increases, its impact diminishes, and the model performance is more likely to be influenced by the network structure. There is a significant decrease in model performance with the SVRDD1K dataset compared to the others due to the fact that the training data size is too small and does not contain sufficient damage instances, which can overfit noise and irrelevant features in the training data, resulting in poor generalization.

**Table 5:** Model performance under sub-datasets with different numbers of training images.

Datasets	Precision	Recall	*F1*-Score	*mAP*@0.5	*mAP*@0.5:0.95
SVRDD6K	0.752	0.671	0.709	0.733	0.417
SVRDD5K	0.719	0.695	0.707	0.733	0.417
SVRDD4K	0.750	0.666	0.706	0.717	0.407
SVRDD3K	0.736	0.659	0.695	0.700	0.387
SVRDD2K	0.708	0.604	0.652	0.648	0.339
SVRDD1K	0.612	0.492	0.545	0.518	0.232

**Fig. 9:**
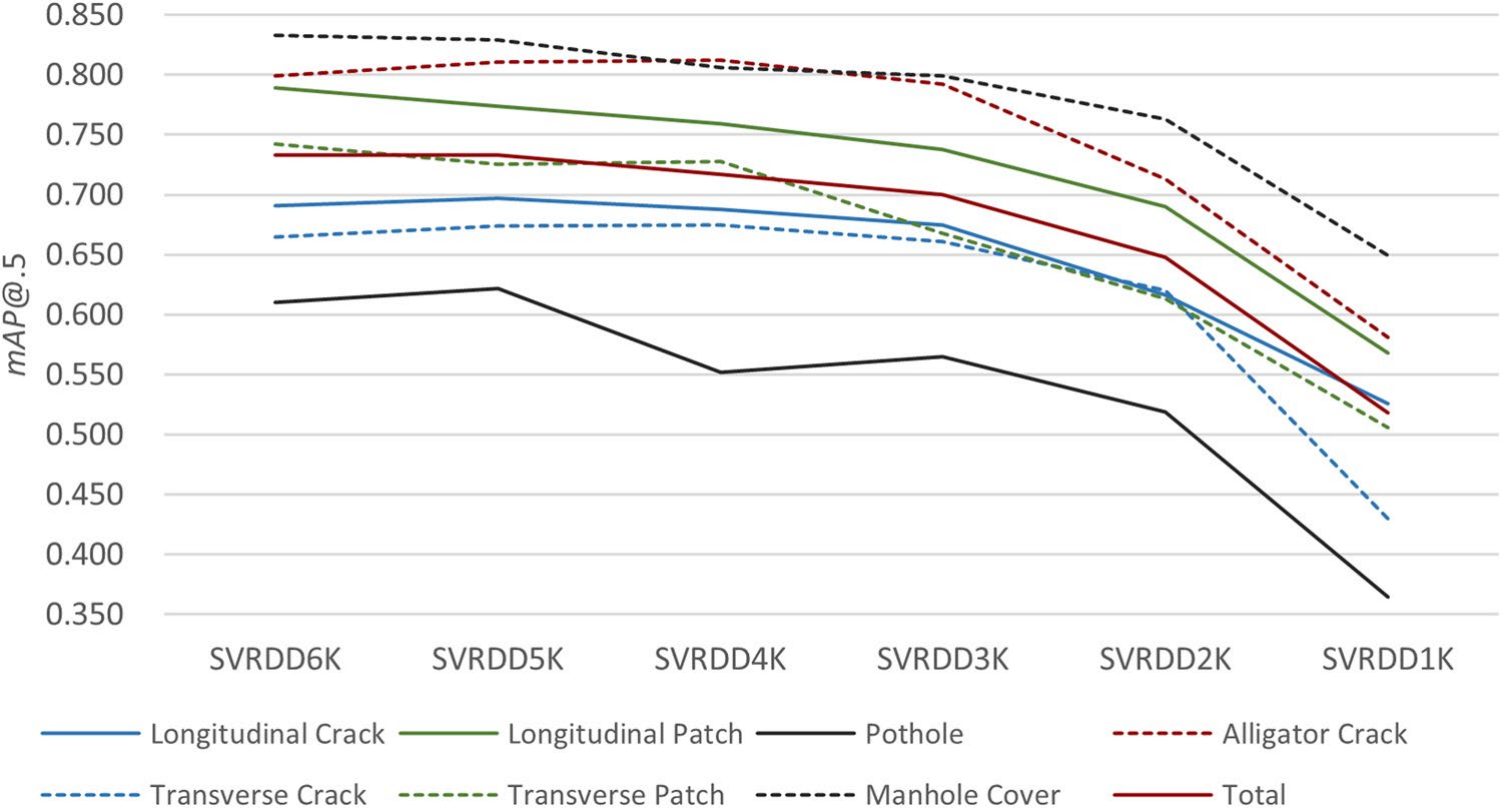
The *mAP*@.5 values of different damage categories with different training set sizes.

The *mAP*@0.5 values for different categories of pavement damages change as the size of the training set varies, with significant differences observed in their respective changes (Fig. 9). In summary, when comparing the *mAP*@0.5 values for different categories of pavement damage: the *mAP*@0.5 for alligator crack is higher than those for patches, which are higher than the *mAP*@0.5 values for cracks, and finally, the *mAP*@0.5 for pothole. Additionally, the *mAP*@0.5 for longitudinal damages are greater than that for transverse ones. The *mAP*@0.5 values of all pavement damage categories generally decrease as the size of the training set decreases. In the decrease of training set sizes from SVRDD6K to SVRDD1K, pothole is most affected with a *mAP*@0.5 decrease of 40.33%. Next, transverse cracks and transverse patches experience a decrease of 35.34% and 31.81% respectively, followed by longitudinal patch and longitudinal crack with decreases of 28.01% and 23.88%. Alligator crack is affected with a decrease of 27.28%. All categories of pavement damage show a substantial drop when the dataset size changes from SVRDD2K to SVRDD1K. Transverse cracks and potholes presented the most significant changes in *mAP*@0.5, with decreases of 30.65% and 29.87% respectively. The remaining *mAP*@0.5 reductions are around 15%. Meanwhile, the *mAP*@0.5 for transverse crack experiences the smallest change when the size transitions from SVRDD6K to SVRDD2K, amounting to only 6.77%. Therefore, when conducting data collection or model training, it is crucial to focus on potholes and transverse cracks as these are the most affected types of pavement damage by the dataset size.

### Performance on removing training sets from different districts

**Fig. 10:**
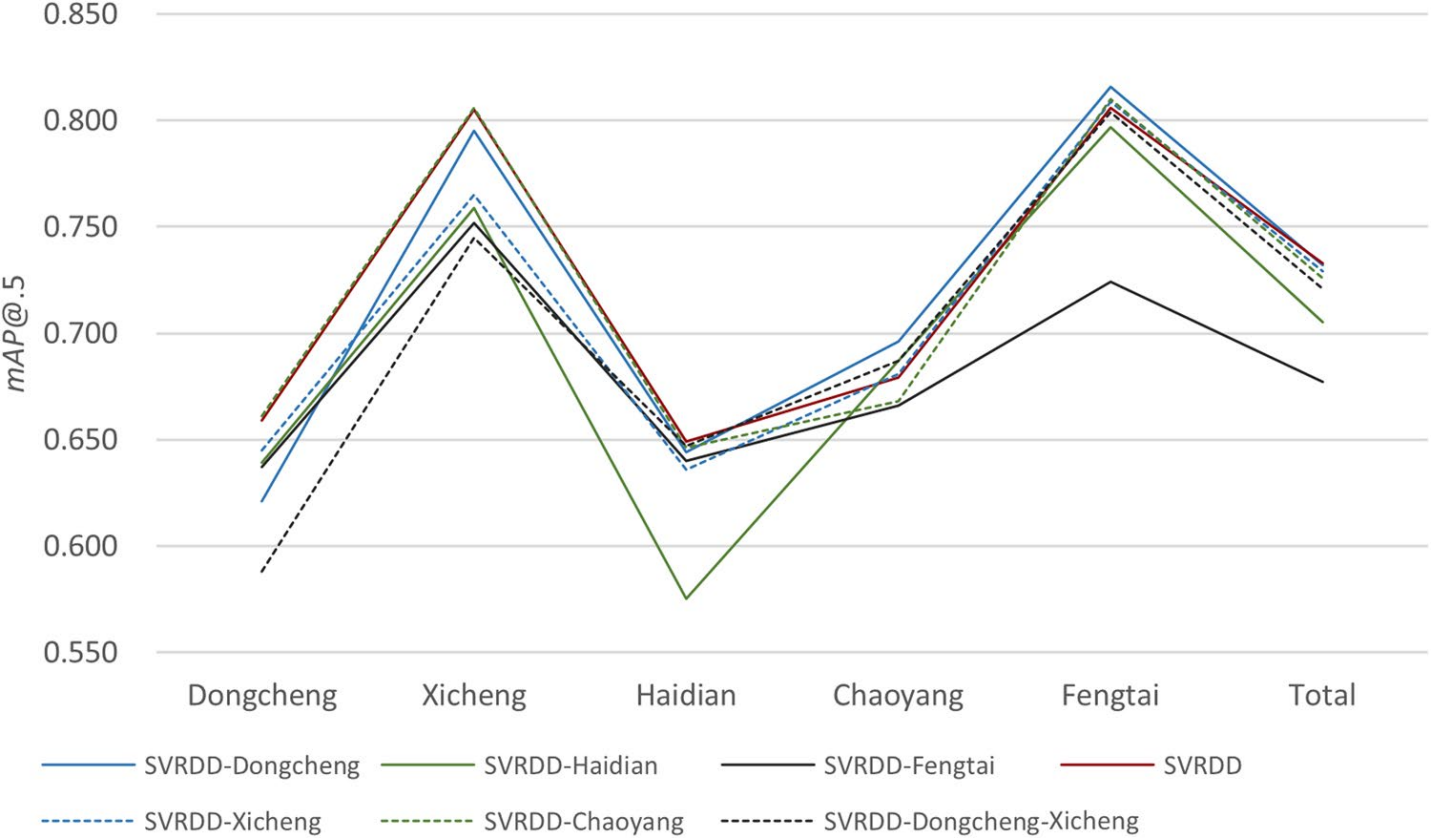
The *mAP*@.5 values of each district after removing training data from each district.

The impact of removing training data from different districts on the model was also investigated. In particular, the validation and testing set in the SVRDD dataset remained consistent, while training images of Dongcheng, Xicheng, Haidian, Chaoyang, and Fengtai were removed to form new datasets. Considering the proximity of Dongcheng District and Xicheng District and that the sum of the image quantities in the two districts is equal to the individual image quantities in the other three districts, we also included the case of simultaneously removing training images from both these two districts. The ‘SVRDD-Dongcheng’ represents the SVRDD dataset with the training data of the Dongcheng District removed and so forth. The YOLOv5 network, which demonstrated the best performance in the comparative experiments, was used for the training and testing. Figure 10 reports the *mAP*@0.5 values for the testing set of each district. The model performance decreased to varying degrees when the training data from different districts was removed. The detection accuracies of Dongcheng and Xicheng districts were the lowest when the training data of these two districts was simultaneously removed out. After the training data of Haidian District was removed, its *mAP*@0.5 value decreased by 0.074, while the decrease of *mAP*@0.5 was insignificant in other cases. This suggests that the image features in the Haidian District were relatively independent because the District is situated across the plain and West Mountain. Removing the training images of Fengtai District had a significant impact on all districts, and thus it had the largest effect on the overall performance. This may be because Fengtai District provides the majority of pothole annotation data in the dataset, which greatly influences the accuracy of pothole detection in all districts, and ultimately affects the overall performance.

## Usage Notes

### Dataset usage

The SVRDD dataset described in this paper is offered to the scientific community for promoting the progress of road damage detection from street view images. It is the first public dataset of street view images for the detection of road damage annotated by trained remote sensing image interpreters and made available publicly. SVRDD can be employed ‘as is’ to train deep learning models for road damage detection. The SVRDD dataset can be downloaded from the link provided. Moreover, the data division strategy used in the technical validation is also provided and users can divide datasets based on their research needs. The users are advised to cite this article and acknowledge the contribution of this dataset to their work.

### Limitations

The SVRDD dataset has some limitations as follows:

- Limited coverage. Although currently covering the five districts of Beijing City, China, the SVRDD dataset spans diverse road types and pavement conditions. It is still less for the vast road network and massive street view data. Pavement damage characteristics may vary from region to region.
- Imbalanced classes. The number of instances in each damage category of the SVRDD dataset is not equal. Potholes were fewer in number in the area studied, so there were significantly fewer instances of potholes than other damages. This could be the reason for the low accuracy of pothole detection (Fig. 9).

### Supported extension

The applicability of SVRDD can be broadened to encompass other scenarios. Users can select extensions to studies based on their interests, and we first recommend addressing the limitations of the dataset. Potential extensions could include:

- Multi-city imagery. The first aim is to include more cities to expand the dataset. This extension to multi-city street view images is anticipated to enhance diversity and improve pavement damage detection performance in various urban settings.
- Multi-temporal imagery. The image in the SVRDD dataset is the most recent in the timeline for that location. Given the regular updates of street view images, capturing them at different times facilitates a comprehensive analysis of temporal variations in pavement conditions at specific locations. This temporal dimension adds valuable insights for ongoing monitoring and assessment.
- Multi-view imagery. Acknowledging the importance of diverse views, the SVRDD dataset can be used with images from other views. Leveraging domain adaptation techniques, models trained on the SVRDD dataset can effectively be deployed on images captured from varying views. The current domain adaptive object detection is mainly based on teacher-student framework and transformer framework, which is worthwhile to consider further research to be applied to the SVRDD dataset.
- Balancing dataset. Having an imbalanced dataset decreases the sensitivity of the model towards minority classes. To handle class imbalance problem, images of fewer number of damage categories can be added in. Various techniques can also be employed, such as data augmentation, resampling techniques, synthetic minority over-sampling technique, etc.
- Additional annotations. Beyond its primary focus, SVRDD images can be annotated for various applications, such as identifying road traffic signs, road markings, congestion detection, and more. This expanded annotation approach adds versatility and utility to the dataset for diverse applications.

## Data Availability

The software used for labelling the pavement damage object bounding boxes is *LabelImg*, provided by https://github.com/HumanSignal/labelImg. In fairness to test the usefulness of the dataset, the framework used for training and testing object detection algorithms in the technical validation is *MMDetection*, provided by https://github.com/open-mmlab/mmdetection.

## References

[1] Chen X, Zhang X, Li J, Ren M, Zhou B (2022). A New Method for Automated Monitoring of Road Pavement Aging Conditions Based on Recurrent Neural Network. IEEE Trans. Intell. Transp. Syst..

[2] Pan Y (2017). Mapping asphalt pavement aging and condition using multiple endmember spectral mixture analysis in Beijing. China. J. Appl. Remote Sens..

[3] Radopoulou SC, Brilakis I (2017). Automated Detection of Multiple Pavement Defects. J. Comput. Civil. Eng..

[4] Shi Y, Cui L, Qi Z, Meng F, Chen Z (2016). Automatic Road Crack Detection Using Random Structured Forests. IEEE Trans. Intell. Transp. Syst..

[5] Cui, L., Qi, Z., Chen, Z., Meng, F. & Shi, Y. Pavement Distress Detection Using Random Decision Forests. In *Proc. Int. Conf. Data Science (ICDS)*, 95-102 (2015).

[6] Zou Q, Cao Y, Li Q, Mao Q, Wang S (2012). CrackTree: Automatic crack detection from pavement images. Pattern Recognit. Lett..

[7] Zou Q (2019). DeepCrack: Learning Hierarchical Convolutional Features for Crack Detection. IEEE Trans. Image Process..

[8] Liu Y, Yao J, Lu X, Xie R, Li L (2019). DeepCrack: A deep hierarchical feature learning architecture for crack segmentation. Neurocomputing.

[9] Yang F (2020). Feature Pyramid and Hierarchical Boosting Network for Pavement Crack Detection. IEEE Trans. Intell. Transp. Syst..

[10] Xu Z (2022). Pavement crack detection from CCD images with a locally enhanced transformer network. Int. J. Appl. Earth Obs. Geoinf..

[11] Guo F, Qian Y, Liu J, Yu H (2023). Pavement crack detection based on transformer network. Autom. Constr..

[12] Eisenbach, M. *et al*. How to get pavement distress detection ready for deep learning? A systematic approach. In *Proc. Int. Joint Conf. Neural Netw. (IJCNN)*, 2039-2047 (2017).

[13] Stricker, R., Eisenbach, M., Sesselmann, M., Debes, K. & Gross, H. M. Improving Visual Road Condition Assessment by Extensive Experiments on the Extended GAPs Dataset. In *Proc. Int. Joint Conf. Neural Netw. (IJCNN)*, 1-8 (2019).

[14] Stricker, R. *et al*. Road Surface Segmentation - Pixel-Perfect Distress and Object Detection for Road Assessment. In *Proc. IEEE 17th Int. Conf. Autom. Sci. Eng. (CASE**)*, 1789-1796 (2021).

[15] Maeda H, Sekimoto Y, Seto T, Kashiyama T, Omata H (2018). Road Damage Detection and Classification Using Deep Neural Networks with Smartphone Images. Comput.-Aided Civil Infrastruct. Eng..

[16] Maeda H, Kashiyama T, Sekimoto Y, Seto T, Omata H (2021). Generative adversarial network for road damage detection. Comput.-Aided Civil Infrastruct. Eng..

[17] Arya D, Maeda H, Ghosh SK, Toshniwal D, Sekimoto Y (2021). RDD2020: An annotated image dataset for automatic road damage detection using deep learning. Data Brief.

[18] Arya, D., Maeda, H., Ghosh, S. K., Toshniwal, D. & Sekimoto, Y. RDD2022: A multi-national image dataset for automatic Road Damage Detection. Preprint at https://arxiv.org/abs/2209.08538 (2022). 10.1016/j.dib.2021.107133 PMC8166755 34095382

[19] Arya, D. *et al*. Global Road Damage Detection: State-of-the-art Solutions. In *Proc. IEEE Int. Conf. Big Data (Big Data)*, 5533-5539 (2020).

[20] Anguelov D (2010). Google Street View: Capturing the World at Street Level. Computer.

[21] Yao Y (2019). A human-machine adversarial scoring framework for urban perception assessment using street-view images. Int. J. Geogr. Inf. Sci..

[22] Fan Z, Zhang F, Loo BPY, Ratti C (2023). Urban visual intelligence: Uncovering hidden city profiles with street view images. Proc. Natl. Acad. Sci..

[23] Yao Y (2023). Extracting the pickpocketing information implied in the built environment by treating it as the anomalies. Cities.

[24] Chacra, D. B. A. & Zelek, J. S. Fully Automated Road Defect Detection Using Street View Images. In *Proc. 14th Conf. Comput. Robot Vis. (CRV)*, 353-360 (2017).

[25] Ma, K., Hoai, M. & Samaras, D. Large-scale Continual Road Inspection: Visual Infrastructure Assessment in the Wild. In *Proc. British Mach. Vis. Conf*. (2017).

[26] Majidifard H, Jin P, Adu-Gyamfi Y, Buttlar WG (2020). Pavement Image Datasets: A New Benchmark Dataset to Classify and Densify Pavement Distresses. Transp. Res. Record.

[27] Lei X, Liu C, Li L, Wang G (2020). Automated Pavement Distress Detection and Deterioration Analysis Using Street View Map. IEEE Access.

[28] Zhang M, Liu Y, Luo S, Gao S (2020). Research on Baidu Street View Road Crack Information Extraction Based on Deep Learning Method. J. Phys.: Conf. Ser..

[29] Maniat M, Camp CV, Kashani AR (2021). Deep learning-based visual crack detection using Google Street View images. Neural Comput. Appl..

[30] Shu Z, Yan Z, Xu X (2021). Pavement Crack Detection Method of Street View Images Based on Deep Learning. J. Phys.: Conf. Ser..

[31] Ren M, Zhang X, Chen X, Zhou B, Feng Z (2023). YOLOv5s-M: A deep learning network model for road pavement damage detection from urban street-view imagery. Int. J. Appl. Earth Obs. Geoinf..

[32] Baidu Maps. Baidu Maps. https://map.baidu.com. Accessed March 15, 2024.

[33] Baidu Maps. Baidu Maps Open Platform Terms of Service. https://lbsyun.baidu.com/index.php?title=open/law. Accessed March 15, 2024.

[34] Ren M, Zhi X, Wei Y, Zhang X (2023). Zenodo.

[35] Ren S, He K, Girshick R, Sun J (2017). Faster R-CNN: Towards Real-Time Object Detection with Region Proposal Networks. IEEE Trans. Pattern Anal. Mach. Intell..

[36] Cai, Z. & Vasconcelos, N. Cascade R-CNN: Delving Into High Quality Object Detection. In *Proc. IEEE Conf. Comput. Vis. Pattern Recognit. (CVPR)*, 6154-6162 (2018).

[37] Zhang, H., Chang, H., Ma, B., Wang, N. & Chen, X. Dynamic R-CNN: Towards High Quality Object Detection via Dynamic Training. In *Proc. Eur. Conf. Comput. Vis. (ECCV)*, 260-275 (2020).

[38] Lin, T.-Y., Goyal, P., Girshick, R., He, K. & Dollár, P. Focal Loss for Dense Object Detection. In *Proc. IEEE Conf. Comput. Vis. Pattern Recognit. (CVPR)*, 2980-2988 (2017). 10.1109/TPAMI.2018.2858826 30040631

[39] Tian, Z., Shen, C., Chen, H. & He, T. FCOS: Fully Convolutional One-Stage Object Detection. In *Proc. IEEE/CVF Int. Conf. Comput. Vis. (ICCV)*, 9627-9636 (2019).

[40] Zhang, S., Chi, C., Yao, Y., Lei, Z. & Li, S. Z. Bridging the Gap Between Anchor-Based and Anchor-Free Detection via Adaptive Training Sample Selection. In *Proc. IEEE/CVF Conf. Comput. Vis. Pattern Recognit. (CVPR)*, 9759-9768 (2020).

[41] Redmon, J. & Farhadi, A. YOLOv3: An Incremental Improvement. Preprint at https://arxiv.org/abs/1804.02767 (2018).

[42] Chen, Q. *et al*. You Only Look One-level Feature. In *Proc. IEEE/CVF Conf. Comput. Vis. Pattern Recognit. (CVPR)*, 13039-13048 (2021).

[43] Jocher G (2022). Zenodo.

[44] Ge, Z., Liu, S., Wang, F., Li, Z. & Sun, J. YOLOX: Exceeding YOLO Series in 2021. Preprint at https://arxiv.org/abs/2107.08430 (2021).

[45] Chen, K. *et al*. MMDetection: Open MMLab Detection Toolbox and Benchmark. Preprint at https://arxiv.org/abs/1906.07155 (2019).

